# Potential advantage of student-run clinics for diversifying a medical school class

**DOI:** 10.3352/jeehp.2012.9.8

**Published:** 2012-05-25

**Authors:** Chris N. Gu, Jane A. McElroy, Blake C. Corcoran

**Affiliations:** Department of Family and Community Medicine and Missouri University Research Reactor, University of Missouri-Columbia School of Medicine, Columbia, MO, USA.

**Keywords:** Medical student, Clinic, Evaluation, Medical education

## Abstract

The purpose of this study was to evaluate the influence of a student-run clinic on the diversification of a medical student class. We distributed a two-page, 20-item, paper survey to students of the University of Missouri School of Medicine (MU SOM) class of 2015 in July of 2011. The survey gathered information on general demographics, opinions on the importance of medical education opportunities, and opinions on the importance of medical school characteristics in applying to and attending MU SOM. A total of 104 students responded to the survey. A majority of the students identified the MedZou Community Health Clinic, a student-run, free health clinic affiliated with MU SOM, and simulated-patient encounters as important educational experiences (81% and 94%, respectively). More than half of the self-identified "non-white" students reported MedZou as an important factor in their choice to apply to (60%; 95% confidence interval [CI], 32 to 88) and attend (71%; 95% CI, 44 to 98) MU SOM, over half of the females reported MedZou as important in their choice to apply (59%; 95% CI, 43 to 76) and attend (57%; 95% CI, 40 to 74), and over half of non-Missouri residents reported MedZou as important in their choice to apply (64%; 95% CI, 36 to 93) and attend (71%; 95% CI, 44 to 98). According to the above results, it can be said that students clearly value both MedZou and simulated-patient encounters as important educational experiences. Women, minorities, and non-Missouri residents value MedZou more highly than their peers who are First Year Medical Students who are Missouri residents, suggesting that MedZou may provide a promising opportunity to advance diversity within MU SOM. These results highlight the need for additional research to further explore MedZou's potential to enhance the recruitment of a diverse medical student class.

The 21st century has seen health care reform escalate to the heights of national attention in the US. Proposed and enacted legislation has focused on expanding eligibility and increasing access to health care nationally [1,2]. Health care organizations have recognized the existence of disparities in health care quality and patient outcomes [3,4], often related to the increasing racial and ethnic diversity of the American population [5]. To abate such disparities, academic authorities have called for increasing the racial and ethnic diversity of the health care workforce, emphasizing the need to produce culturally-competent physicians [6]. These physicians, who possess the knowledge, skills, and behaviors required to provide culturally-effective care to patients from a wide range of backgrounds, will in turn, expand access to higher quality health care for medically underserved populations [6]. The Association of American Medical Colleges (AAMC) has followed suit by making diversity a shared objective and key mission based upon its recognition that diversity enhances the learning for all students and establishes a foundation for more expansive, higher quality care in all communities [7]. Students have self-reported that contact with diverse peers greatly enhances their educational experience and strongly support strengthening or maintaining current affirmative action policies in admissions [8]. Studies have shown that students attending more diverse medical schools rate themselves as better prepared to care for racial and ethnic minority patients than students at less diverse schools [9]. This association appears to be mediated by more positive interaction and perspective sharing among individuals from different backgrounds within medical schools. Medical schools have placed similar value on diversity by incorporating goals for diversity enhancement into mission statements [10], creating programs focused on meeting the health care needs of underserved and minority populations both within the US and internationally [11-14], and producing more culturally-competent physicians. One way of reaching these patients has been through the development of student-run clinics (SRCs) [12,15]. In recent decades, SRCs have emerged across the country to provide health care to the underserved and uninsured: populations composed mostly of minority patients [15]. A recent literature review showed that at least 49 medical schools operate at least 110 SRCs [16]. The Society of Student-Run Free Health Clinics, the largest organization and collective voice of SRCs in the US, currently has a membership of 87 registered clinics in 29 states. While research has been conducted on the value of SRCs in medical-student education [16] and the impact of SRCs on health outcomes for patients with diseases including diabetes [17], hypertension [18], and mental illness [19], no study, to our knowledge, has explored how SRCs impact medical school diversity, recruitment, or matriculation.

The purpose of this study was to evaluate the influence of a student-run clinic on the recruitment and matriculation of a diverse medical student class. How SRCs are used in the recruitment of prospective medical students and how these students value SRCs are questions that could have implications for the use of SRCs in the targeted recruitment of specific student populations: students that will increase diversity within a school of medicine.

We distributed a two-page, 20-item paper survey to students of the University of Missouri School of Medicine (MU SOM) class of 2015 at the end of an informational session on extracurricular activities during orientation week. Completed surveys were collected the same day. This project was approved by the University of Missouri Health Sciences Institutional Review Board. The survey consisted of five main categories of questions: general demographics, intended type and place of medical specialty, opinions about the importance of medical education opportunities, and the importance of medical school characteristics in applying to and attending MU SOM. General demographic information included age (continuous), sexual orientation (six choices: gay, lesbian, bisexual, heterosexual or straight, don't know/not sure, and other), race (five choices: Black or African American, Asian, White, American Indian or Alaska Native, and Native Hawaiian or Other Pacific Islander), gender (two choices: man and woman), marital status (five choices: single, married or in a married-like partnership, divorced, separated or widowed), Missouri-resident status (dichotomous: yes or no), transgender or transsexual status (three choices: yes transgender, yes transsexual and no), and ethnicity as hispanic or latino/a (dichotomous: yes or no). Questions regarding intended medical specialties and geographic areas of practice were open-ended and included a checkbox for "I have no idea". Educational experiences offered at MU SOM (simulated-patient encounters, the STEP program which partners seniors in the community with medical students, rural medicine opportunities and experiences, MedZou Clinic, and research opportunities between preclinical years and within the MD/PhD program) were rated on a five-point Likert-type scale from very important to not at all important. Factors that influenced the students' decision to apply to or attend MU SOM (10 characteristics: general reputation of MU, research reputation of MU, interviews and meetings with faculty and/or administrators, advice of students, geographic location of MU, financial considerations, MedZou Clinic opportunities, Problem Based Learning (PBL) curriculum, advice of parents or family members, and advice of premedical advisors) were rated on a five-point Likert-type scale from "very important" to "not at all important". In addition, the option "no idea/don't know" was available.

We used descriptive statistics (frequencies, mean, and 95% confidence intervals [CIs]) to describe responses that measured demographic characteristics, educational experiences, and factors that influenced students' decisions to apply to and attend MU SOM.

We grouped the two responses highest on the Likert-type importance scale ("very important" and "important") into one category as "important." Participants who checked more than one answer choice for race were categorized as "multiracial", and we reported all students who identified with any non-white answer choice as "non-white."

Two MU SOM pre-admittance programs guarantee medical school admission to exemplary high school and undergraduate students. These students underwent a different application and interview process. Therefore, all students who responded as applying to only one medical school (n=23) were excluded from data analysis involving factors that influenced students' decision to apply to or attend MU SOM.

A total of 104 students participated in the study (100% participation proportion). The ages of respondents ranged from 20 to 36 years, with an average of 23.5 years. Of the respondents, 52 reported as male, 52 as female, and none reported as transgender or transsexual. Less than10 students self-identified as a sexual or gender minority. The sample included 85 white students and 19 non-white students. Additionally, 88 students classified themselves as a Missouri resident and 16 as a non-Missouri resident. Of the five anticipated medical education experiences, a majority of students identified simulated-patient encounters and MedZou Clinic (94% and 81%, respectively) as important. The other three experiences had substantially fewer students considering them important (range, 34% to 42%). Of the ten factors in the decision to apply to MU SOM, the majority of students reported the PBL curriculum, financial considerations, and the general reputation of MU SOM important (86%, 84%, and 77% of students, respectively). The majority of students reported these same three factors important in their decision to attend MU SOM (89%, 79%, and 78%, respectively).

While approximately half of the students ranked MedZou Clinic important in their decision to apply to (48%) and attend (53%) MU SOM, substantial differences were observed when these data were evaluated according to race, gender, and Missouri-resident status. For students who self-identified as "white", less than half reported MedZou as important in their decision to apply to (45%; 95% CI, 33 to 58) and attend (48%; 95% CI, 36 to 61) MU SOM. In contrast, more than half of "non-white" students reported MedZou important to their choice to apply (60%; 95% CI, 32 to 88) and attend (71%; 95% CI, 44 to 98). While less than half of the males considered MedZou important in their decision to apply to (39%; 95% CI, 24 to 54) and attend (49%; 95% CI, 33 to 64) MU SOM, over half of the females reported MedZou important in their decision to apply (59%; 95% CI, 43 to 76) and attend (57%; 95% CI, 40 to 74). Approximately half of Missouri residents considered MedZou important in their decision to apply to (46%; 95% CI, 34 to 59) and attend (50%; 95% CI, 37 to 63) MU SOM, but two-thirds of non-Missouri residents considered MedZou important in their decision to apply (64%; 95% CI, 36 to 93) and attend (71%; 95% CI, 44 to 98).

Valuing diversity in the medical school admission process has important implications for our health care system. Namely, a diverse student body leads to improved quality of medical education, access to health care for underserved populations, and medical and public health research [20]. Many of these same beliefs are echoed by national health care organizations [7], medical schools [10], and students themselves [8]. Our study found that students from diverse backgrounds, specifically non-whites, females and non-Missouri residents, placed greater importance on MedZou (a student-run, free health clinic affiliated with the MU SOM) than their peers when making their decision to apply to and attend MU SOM.

While MedZou has been recognized for its educational value by the MU SOM and the surrounding community [21-26], its potential as a recruitment tool has yet to be fully realized. MU SOM's definition of diversity includes differing genders, religious beliefs, sexual orientations, racial-ethnic backgrounds, national origins and socioeconomic classes. MU SOM's recognition that the enrichment inclusion provides to the art and practice of medicine [10] and their goal of building a diverse student community may be more fully realized through an increased emphasis on MedZou during the recruitment process. If further substantiated, this emphasis may lead to the increased cultural competency of medical students and future physicians who will be better equipped with the tools to provide culturally-effective care to patients from diverse backgrounds. Our study reveals the relative significance of MedZou and its anticipated educational value in a student's decision to apply to and attend MU SOM. When comparing factors that influence this decision, fewer surveyed students considered MedZou as important as the PBL curriculum, general reputation of MU SOM, or financial considerations. Possible explanations are that students may believe the curriculum and reputation of a medical school leads to better learning and preparedness for post-medical school training, and may be concerned about the increasing financial burden of professional education. While rating the factors that influence the decision to apply to and attend MU SOM can be thought of as anticipatory in determining which factors are of importance in selecting a medical school, asking students to consider educational experiences offered at MU SOM can be thought of as anticipating what is important to the students' medical education after admittance. Interestingly, the majority of students considered MedZou and simulated-patient encounters to be the two most important educational experiences offered. Similarities exist between these experiences, as both offer students the opportunity to translate their education in the classrooms and lecture halls into clinically-relevant encounters. This may mean that students believe in the importance of seeing real or simulated patients prior to their clinical years of medical education.

One limitation of our study was a small sample size, particularly when analyzing subgroups, and while the data showed substantial differences between subgroups of students, these were not statistically significant. If these differences between subgroups are reproduced across a larger medical student population through the design and implementation of a multi-school, multi-clinic study, it may further validate the impact of SRCs on the recruitment and matriculation of diverse students and advancement of diversity in the health care system.

In conclusion, first-year medical school students clearly indicated the importance of an SRC as an anticipated educational experience. While only half of the surveyed students considered MedZou important in their decision to apply to or attend MU SOM, a higher proportion of women, minorities, and non-Missouri residents considered MedZou important in their decision to apply and attend compared to their counterparts. This finding illustrates the unintended, yet promising, role that an SRC may play in the recruitment and advancement of diversity within MU SOM and the health care system at large. In order to substantiate these findings, a larger, multi-school, multi-clinic study is recommended.

## References

[1] (2001). Bipartisan Patient Protection Act, S. 1052 [Internet]. 107th Congress of the USA.

[2] (2010). Patient Protection and Affordable Care Act, Public Law 111-148 [Internet]. 111th Congress of the USA.

[3] US Department of Health and Human Services (2000). Healthy people 2010: understanding and improving health.

[4] Smedley BD, Stith AY, Nelson AR (2003). Unequal treatment: confronting racial and ethnic disparities in health care.

[5] (2004). Projected population of the United States, by race and Hispanic origin: 2000 to 2050 [Internet]. US Census Bureau.

[6] Cohen JJ, Gabriel BA, Terrell C (2002). The case for diversity in the health care workforce. Health Aff (Millwood).

[7] Coleman AL, Palmer SR, Winnick SY (2008). Roadmap to diversity: key legal and educational policy foundations for medical schools.

[8] Whitla DK, Orfield G, Silen W, Teperow C, Howard C, Reede J (2003). Educational benefits of diversity in medical school: a survey of students. Acad Med.

[9] Saha S, Guiton G, Wimmers PF, Wilkerson L (2008). Student body racial and ethnic composition and diversity-related outcomes in US medical schools. JAMA.

[10] (2011). The University of Missouri School of Medicine. Diversity and inclusion [Internet].

[11] Mann S (2011). Medical schools focus on meeting health care needs of growing Hispanic population [Internet].

[12] Pi R (1995). The Asian clinic at UC Davis: serving a minority population for two decades. JAMA.

[13] Wee LE, Yeo WX, Tay CM, Lee JJ, Koh GC (2010). The pedagogical value of a student-run community-based experiential learning project: the Yong Loo Lin School of Medicine Public Health Screening. Ann Acad Med Singapore.

[14] Liang En W, Koh GC, Lim VK (2011). Caring for underserved patients through neighborhood health screening: outcomes of a longitudinal, interprofessional, student-run home visit program in Singapore. Acad Med.

[15] Simpson SA, Long JA (2007). Medical student-run health clinics: important contributors to patient care and medical education. J Gen Intern Med.

[16] Meah YS, Smith EL, Thomas DC (2009). Student-run health clinic: novel arena to educate medical students on systems-based practice. Mt Sinai J Med.

[17] Ryskina KL, Meah YS, Thomas DC (2009). Quality of diabetes care at a student-run free clinic. J Health Care Poor Underserved.

[18] Zucker J, Gillen J, Ackrivo J, Schroeder R, Keller S (2011). Hypertension management in a student-run free clinic: meeting national standards?. Acad Med.

[19] Liberman KM, Meah YS, Chow A, Tornheim J, Rolon O, Thomas DC (2011). Quality of mental health care at a student-run clinic: care for the uninsured exceeds that of publicly and privately insured populations. J Community Health.

[20] Cohen JJ (2003). The consequences of premature abandonment of affirmative action in medical school admissions. JAMA.

[21] Detillier S (2011). MedZou: health care for the poor [Internet].

[22] Moritz K (2012). MedZou clinic celebrates grand opening [Internet].

[23] (2009). MedZou clinic receives Susan G. Komen grant to offer educational outreach [Internet]. The University of Missouri School of Medicine.

[24] (2009). MedZou honored as University's best new organization [Internet]. The University of Missouri School of Medicine.

[25] (2009). MedZou cuts wait time for uninsured patients by providing free primary care [Internet]. The University of Missouri School of Medicine.

[26] (2011). MU student-run clinic offers free services to uninsured patients; volunteers learn how to become future health care providers [Internet].

